# Development and validation of a COVID-19 risk perception scale in Peru

**DOI:** 10.17843/rpmesp.2023.402.12289

**Published:** 2023-06-30

**Authors:** Jhon Alex Zeladita-Huaman, Eduardo Franco-Chalco, Roberto Zegarra-Chapoñan, Ruth Iguiñiz-Romero, Isabel Amemiya-Hoshi

**Affiliations:** 1 Universidad Nacional Mayor de San Marcos, Lima, Peru. Universidad Nacional Mayor de San Marcos Universidad Nacional Mayor de San Marcos Lima Peru; 2 National School of Public Health - ENSAP, Lima, Peru. National School of Public Health - ENSAP Lima Peru; 3 Universidad María Auxiliadora, Lima, Peru Universidad María Auxiliadora Universidad María Auxiliadora Lima Peru; 4 Universidad Peruana Cayetano Heredia, Lima, Peru. Universidad Peruana Cayetano Heredia Universidad Peruana Cayetano Heredia Lima Peru

**Keywords:** Coronavirus Infections, Perception, Psychometrics, Perú

## Abstract

**Objectives.:**

To develop and validate a risk perception scale for COVID-19 (PR-COVID-19-PE) in the Peruvian population.

**Materials and methods.:**

Psychometric cross-sectional study conducted in 2022. In phase 1, in order to design the scale, we carried out a theoretical review and a documentary review of scales, we also used focus groups as well as an expert panel. Phase 2 included expert judgment and a pilot test. A virtual survey was conducted among 678 Peruvian adults during phase 3. A confirmatory factor analysis was carried out as well. We used a correlational analysis (Pearson’s r) with a valid risk perception scale and the COVID-19 fear scale to determine criterion validity.

**Results.:**

The PR-COVID-19-PE has two dimensions (cognitive and emotional) and showed good fit during construct validity (x2/gl=2.34, Comparative Fit Index=0.96, Tucker-Lewis Index=0.96, Root Mean Square Error of Approximation= 0.05 and Standardized Root Mean-Square=0.07) and optimal internal consistency (ώ=0.88). Likewise, the PR-COVID-19-PE showed correlation with another COVID-19 risk perception scale (r=0.70, p< 0.001) and a fear of COVID-19 scale (r=0.41, p<0.001). In addition, it presents metric and scalar invariance by both sex and educational level.

**Conclusions.:**

The PR-COVID-19-PE scale showed adequate reliability and content, construct and criterion validity. It is an instrument that can measure COVID-19 risk perception in similar populations. However, further studies are required for different populations.

## INTRODUCTION

More than 2.9 million deaths were reported worldwide at the end of the coronavirus disease (COVID-19) pandemic ^1^. Vaccines were effective in reducing the severe form of the disease and deaths in the post-pandemic scenario ^(^^2^; therefore, most countries have eliminated mandatory compliance with preventive measures. However, the variants and subvariants that appeared in 2021 continue to spread worldwide, due to changes in their transmissibility, pathogenicity and virulence; even changes regarding symptoms, complications and sequelae ^3^.

On the other hand, risk is neither constant nor can it be reduced to a perception of vulnerability to certain death or of being exempt from harm ^4^^,^^5^. During the pandemic, the population perceived different levels of risk due to the increasing number of deaths, the appearance of new variants, the uncertainty of the sequelae and the long-term effects of this disease ^6^.

Studies conducted at the beginning of the vaccination against COVID-19 reported a decrease in the perception of risk (PR), however, this perception did not go away completely ^2^. Moreover, although the pandemic is at an end, it is still relevant to study the psychological and contextual factors that shaped human behavior in response to a phenomenon that shaped human history, due to the socioeconomic and psychological consequences.

Evidence on how to assess PR is scarce; therefore, in spite of previous research during the influenza A (H1N1) ^7^ and Ebola ^8^ pandemics there is no consensus on how it should be measured. Studies usually assess this construct by using one to three questions ^9^^-^^11^ or in brief self-report questionnaires ^11^^,^^12^. Subsequently, a cross-sectional study carried out in ten countries in Europe, America and Asia validated a multidimensional scale of PR in the face of the COVID-19 pandemic comprising seven items covering cognitive, affective and temporo-spatial dimensions ^13^ and based on the theoretical model of PR in the face of climate change proposed by van der Linden ^14^. Two scales were validated in the Latin American and Caribbean context, one in Cuba ^15^^)^ and the other in Colombia ^16^. Both scales, in addition to considering the cognitive and emotional dimension, included a dimension that evaluates motivations and risk and protection behaviors; aspects that could interfere in the analysis of association with other variables such as applying preventive measures.

However, despite the existence of validated scales to measure PR for COVID-19 in Spanish ^15^^,^^16^, studies in Peru have used scales translated from other languages ^17^, adapted from questionnaires used in other diseases such as cancer ^18^ or by means of a single question ^9^. Another study, carried out on health personnel, used a scale of perception of safety regarding protective measures ^19^. For these reasons, it is necessary to have a scale that really measures PR in the Peruvian population; even more so if it considers that this population is culturally diverse ^20^.

The start of vaccination against COVID-19 led the population to perceive a lower risk of disease severity. However, being able to use a scale to identify how different contextual and psychological factors caused this perception to change from one moment to the next is precisely the aspect that justifies the development of a scale to measure PR. In addition, PR varies according to cultural differences, as well as social, psychological and political factors ^13^ and the intensity of the effects or consequences of SARS-CoV-2 in a given population ^(^^21^. For these reasons, our study aimed at developing and validating a COVID-19 PR scale in the Peruvian population.

**KEY MESSAGES**

Motivation for the study. Risk perception of COVID-19 is a construct that varies according to the characteristics of the population in each geographic area; however, there is no validated scale to measure this construct in the Peruvian population.

Main findings. A COVID-19 risk perception scale composed of two dimensions (cognitive and emotional) was designed and validated using qualitative and quantitative techniques.

Implications. Having a valid and reliable instrument will help identify the variation of risk perception of COVID-19 according to contextual and psychological factors in the Peruvian population.

## MATERIALS AND METHODS

### Study design

We carried out a non-experimental, cross-sectional, instrumental study ^22^ to develop, validate, and determine the psychometric properties of a PR scale for COVID-19 in the Peruvian population. It should be noted that, at the time of the study, Peru was at the beginning of the fourth wave of the COVID-19 pandemic ^23^.

### Instrument

The COVID-19 PR Scale (PR-COVID-19-PE) was developed in this study (Supplementary Material 1).

### Procedures

The COSMIN Checklist (COnsensus-based Standards for the selection of health status Measurement) (https://www.cosmin.nl/) was used to verify the adequacy of the instrument’s validity analysis (Supplementary Material 2).

Our research was organized in three stages based on the ten steps proposed by Muñiz and Fonseca ^24^. The first stage was oriented to designing the scale, for which we used an overall theoretical review of PR and a review of COVID-19 PR in order to stablish a theoretical framework and define the variable, its dimensions and indicators. Then, a panel of nine experts (authors of scientific articles on validation of PR scales before COVID-19 in Latin American countries or who conducted studies on COVID-19 prevention in Peru) validated these definitions and provided suggestions that were included in the following stages of the study.

Subsequently, for the construction of the items, we created six focus groups to explore the social representations on the COVID-19 PR held by different segments of the Peruvian population. The focus groups were made up of regular basic education teachers (primary and secondary level), higher education teachers, higher education students, parents and shopkeepers, who gave their informed consent prior to the session. The statements of the focus group participants were transcribed and coded using the Atlas.Ti v.8 program.

Next, we reviewed documents regarding PR scales before COVID-19 that were validated at the moment ^13^^,^^15^^,^^16^^,^^25^. Data triangulation was carried out considering the statements of the participants in the focus groups, the indicators developed during the theoretical review and the information from the documents reviewed. As a result, we constructed an initial scale, composed of 26 items, its dimensions and response alternatives.

In the second stage of the study, we used experts’ judgements to estimate the content validity, (two psychometric psychologists, a neuroscientific psychologist and two public health research nurses); the documents containing the theoretical definitions and the proposed PR scale together with its rating scale were sent to the experts. The experts’ ratings were consolidated. Then, by using the Content Validity Coefficient concordance index (CVCic) by Hernández Nieto ^26^, we evaluated three criteria: coherence, relevance and clarity.

Simultaneously, we carried out a pilot test to evaluate how to present the scale and to detect items that could be difficult to understand, as well as any type of error. The pilot test was conducted on 41 inhabitants of thirteen cities in Peru, who were invited by telephone to fill a virtual formulary designed in Google Forms that was distributed by WhatsApp. Subsequently, they were asked to complete an additional form to report any difficulty in understanding the items and the response alternatives.

Finally, the third stage was aimed at determining the psychometric properties of the instrument. We initially calculated a minimum sample size of 260, assuming the classic criterion of 10 subjects for each variable ^27^; however, in order to have greater statistical power, we included all 678 participants who answered the survey. The sample was selected by non-random snowball sampling.

Peruvians over 18 years of age were invited to participate between July 5 and 21, 2021. Data collection was carried out with a virtual form designed on the ALLCOUNTED platform (https://www.allcounted.com), which was distributed through social networks and on the website of the National School of Public Health of the Ministry of Health.

### Variable

PR to COVID-19 was defined as the subjective assessment of the probability of contagion and severity of the possible consequences of COVID-19 at the individual, family and global levels. This construct is made up of two dimensions, one cognitive and the other emotional. The definitions of the indicators are presented in Supplementary Material 3.

### Statistical analysis

We evaluated the factor structure of two dimensions by means of a confirmatory factor analysis (CFA) in order to corroborate the hypothesized factor structure of the scale. An exploratory factor analysis was not carried out because a theoretical structure was defined from the conceptual framework and the qualitative analysis; therefore, the CFA was a better choice ^28^^,^^29^. The least squares estimator of weighted means and variances was used due to the ordinal nature of the instrument. To identify the absolute fit of the model, we assessed whether the chi-square ratio between the degrees of freedom (X^2^/ gl) was less than 3. Similarly, to evaluate the relative fit, we assessed whether the Comparative Fit Index (CFI) and Tucker-Lewis Index (TLI) indicators were greater than 0.95, as well as whether the Root Mean Square Error of Approximation (RMSEA) indicator was equal to or less than 0.05 and if the Standardized Root Mean-Square (SRMR) indicator was equal to or less than 0.08 ^30^^-^^32^.We considered eliminating items that did not have a significant factor load or those with very low levels. Similarly, we performed an invariance analysis according to sex and educational level to compare the configural, metric and scalar invariance. For this criterion, changes less than or equal to 0.01 in CFI ^33^, and changes less than or equal to 0.015 in RMSEA ^34^ were considered to have reached the level of invariance.

Internal consistency was determined with the McDonald omega coefficient (ώ). To verify the criterion validity of the PR-COVID-19-PE, two questionnaires were used as contrast measures. First, it was contrasted with a validated questionnaire that measures PR before the COVID-19 ^13^, which was selected because it uses the theoretical model on which the scale was constructed ^14^, however, due to the low reliability (ώ=0.53), item 6 was eliminated as it showed no correlation with the other items of the instrument; thus, achieving an increase in the reliability index (ώ=0.68 of the survey). The second contrast with the COVID-19 fear questionnaire ^35^ showed high reliability (ώ=0.93). Both instruments were correlated with PR-COVID-19-PE, by using Pearson’s r correlation coefficient. We applied the Little test, which contrasts the null hypothesis of missing values completely at random (MCR), and found insufficient evidence to reject that fact that the data loss was missing completely at random, so we applied multiple imputation with 20 databases and the Predictive Mean Matching (PMM) algorithm. We reviewed quantile-quantile graphs and Shapiro-Wilk tests to determine the level of normality of the variables, and found that the distribution of the data meets sufficient criteria to consider that they come from a normal distribution. Therefore, we used parametric tests to identify the associations of concurrent validity. All statistical analyses were performed in R v 4.2.1 software and its interface R Studio v 2021.0.

### Ethical Aspects

The article is derived from the study entitled: “*Percepción de riesgo y prácticas de medidas preventivas ante la COVID-19 en docentes de Educación Básica Regular, Perú, 2022: Un estudio mixto*” which was approved by the Ethics and Research Committee of the María Auxiliadora University (Act n.° 004-2022) and registered (code EI00000002729) in the Platform for Health Research Projects (PRISA) of the National Institute of Health (INS). The participants gave their informed consent prior to participation All data were anonymized.

## RESULTS

The focus groups included 29 participants from 11 Peruvian cities, of whom 17 were women (58.6%) and the average age was 41.07 years (SD=14.19). Of the participants, 58.6% reported having completed higher education, 13.7% had incomplete higher education, and 27.6% had only completed secondary school. Likewise, 41 people participated in the pilot test, 70% of whom were female and 36.6% lived in Lima.

### Scale validity

The CVCic obtained for each of the items evaluated by expert judgment can be found in Table 1, which shows that most of them obtained values greater than 0.90. Likewise, the CVCic obtained for the coherence, relevance and clarity criteria were greater than 0.90.

**Table 1:** Content validity coefficients for the criteria of coherence, relevance and clarity per item of the initial scale of risk perception to COVID-19.

Item	Coherence	Relevance	Clarity
1	0.99	0.99	0.96
2	0.96	0.99	0.96
3	0.99	0.99	0.96
4	0.83	0.75	0.96
5	0.88	0.83	0.99
6	0.99	0.92	0.83
7	0.99	0.96	0.99
8	0.96	0.92	0.96
9	0.96	0.92	0.99
10	0.92	0.88	0.99
11	0.99	0.96	0.99
12	0.96	0.92	0.99
13	0.99	0.99	0.99
14	0.99	0.99	0.99
15	0.96	0.92	0.99
16	0.75	0.71	0.92
17	0.83	0.92	0.99
18	0.88	0.92	0.99
19	0.83	0.96	0.99
20	0.92	0.92	0.99
21	0.99	0.99	0.99
22	0.99	0.99	0.99
23	0.96	0.96	0.96
24	0.96	0.96	0.96
25	0.99	0.99	0.99
26	0.92	0.88	0.99
CVCic per criterion	0.94	0.93	0.98

CVCic = content validity coefficient concordance index.

During the pilot test, we determined that the average time to apply the questionnaire was 12 min. Of the 41 participants in the pilot test, 34 agreed to complete the second form by means of an interview. At this stage, only 6 of the 34 reported having some difficulty in answering the items.

Finally, all the items that reported CVCic below 0.90 by the expert judgment and those that reported difficulties in the pilot test were evaluated. During this evaluation, we reformulated 9 items (2, 3, 7, 8, 9, 10, 16, 17 and 20), 3 items were changed to another dimension (17, 18 and 19) and 3 items were eliminated (4, 5 and 6); thus, the scale consisted of 23 items.

### Confirmatory factor analysis

The virtual survey for the CFA was answered by 780 participants, of which five were excluded for quality control and 97 because they answered only the first part of the questionnaire. Of the total of 678 participants, most were women (67.0%), with an average age of 34.5 years (SD= 12.41). Of the sample, 50.8% reported having at least higher education, 24.3% reported having incomplete higher education, while the rest of the participants reported lower education levels. Regarding previous experience with COVID-19, 59.2% of the participants reported having been previously infected and 2.5% reported having a diagnosis of COVID-19 at the time of responding to the survey. On the other hand, 34.3% of the participants reported having lost a close person or family member to COVID-19.

**Figure 1:**
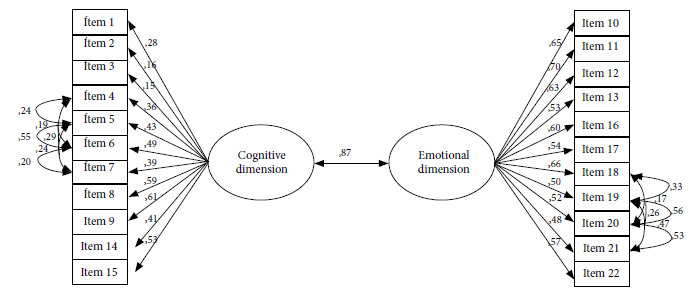
Factor model for the COVID-19 risk perception scale.

Initially, when considering all items, we obtained the following fit indicators: X^2^/gl=3.61, CFI=0.92, TLI= 0.91, RMSEA=0.07 and SRMR=0.09. Then, we detected that the reverse coding item “If I get COVID-19 it will be like a simple flu or something temporary, then I can continue with my life” did not have a significant factor load that would allow an adequate adjustment of the hypothetical model used, so this item was eliminated from the model. Similarly, the residual correlations of the items with similar wording were left free (e.g., items 4, 5, 6 and 7; and items 19, 20, 21, 22). Finally, the fit indicators without considering the reverse coding item were: X^2^/gl=2.34, CFI=0.96, TLI=0.96, RMSEA=0.05 and SRMR = 0.07. The final factorial model tested is shown in Figure 1, which consisted of 22 items.

Table 2 shows the averages and standard deviations for each of the PR-COVID-19-PE items. Overall, item 17 obtained the highest mean score (M=4.21, SD=0.69), while item 4 obtained the lowest mean score (M=3.15, SD=0.90). Given that all items were measured on a scale of 1 to 5, we found that the averages of all items were higher than 3, this could indicate that the people who participated in the study had a risk perception above the average value of three.

**Table 2:** Average and standard deviation by items of the COVID-19 Risk Perception Questionnaire.

	M	SD
1. I am aware that I could still be infected with COVID-19 and its variants.	4.20	0.99
2. If I complete the vaccination scheme, I will avoid becoming infected with COVID-19 and its variants.	3.18	1.25
3. I feel protected from COVID-19 and its variants if I comply with the prevention protocols.	3.96	1.00
4. What do you consider to be the level of risk that an apparently healthy family member or friend has of becoming infected with COVID-19?	3.15	0.96
5. What do you consider to be the level of risk that a vulnerable family member or friend has of becoming infected with COVID-19?	4.13	0.90
6. What do you consider to be the level of risk that an older adult family member or friend has of becoming infected with COVID-19?	4.19	0.83
7. What do you consider to be the level of risk that a child has of becoming infected with COVID-19?	3.36	1.14
8. The risk of a new wave of COVID-19 infection reaching Peru is imminent or high.	4.05	0.83
9. The risk of new COVID-19 variants appearing in the world is imminent or high.	4.07	0.83
10. I am afraid of contracting COVID-19 and its variants.	3.66	1.05
11. I am afraid that one of my family members will be infected with COVID-19 and its variants.	4.02	0.89
12. If I or any of my family members were infected with COVID-19, I would be afraid and fear death.	3.71	1.01
13. If I am infected with COVID-19 and its variants, I would be at risk of sequelae.	3.84	0.86
14. If I become infected with COVID-19 and its variants, I could become seriously ill and will require hospitalization.	3.36	1.02
15. Becoming ill or having sequelae from COVID-19 could have consequences on my work performance or daily activities.	3.67	0.93
16. COVID-19 generates pain for the family and concern for the children and relatives.	4.21	0.69
17. Getting COVID-19 will affect my social life.	3.57	1.02
18. To what extent are you concerned about becoming infected or any of your family members becoming infected with COVID-19 and its variants?	3.83	1.07
19. To what extent do you consider that a new wave of COVID-19 infections will affect Peru’s economic stability (food shortages, increased poverty and unemployment)?	4.08	0.90
20. To what extent do you consider that a new wave of COVID-19 infections will cause a crisis in the Peruvian health system (collapse of hospitals or shortage of medicines)?	4.08	0.94
21. To what extent do you consider that a new wave of COVID-19 infections will affect the mental health of Peruvians (increased anxiety, fear and dread)?	4.02	0.90
22. A new wave of COVID-19 infections will cause a worldwide economic crisis.	4.14	0.75

M: mean, SD: standard deviation.

The factor structure resulting from the confirmatory model, the factor loadings and their respective significance are presented in Figure 1. All items of the cognitive dimension of PR-COVID-19-PE have statistically significant factor loadings (p<0.001), except for item 2 (p=0.001) and item 3 (p=0.003). However, it is important to highlight that items 1, 2 and 3 have factor loadings with low effect sizes (0.28, 0.16 and 0.15, respectively). This indicates that the three items have a significant loading, but are not as strongly correlated with the cognitive dimension; however, they were kept because they may explain some characteristics of the cognitive dimension that other items could not. All the other items of this dimension had factor loadings higher than 0.30, which shows that they have good correlation with the cognitive dimension factor. All the items of the emotional dimension have factor loadings greater than 0.30 and are statistically significant (p<0.001). Furthermore, a very strong correlation was found between both dimensions of the scale (r=0.87, p<0.001). Finally, we confirmed that PR-COVID-19-PE has an excellent internal consistency coefficient (ώ=0.88).

Table 3 presents the results of the invariance analyses according to sex and educational level. We compared subjects with less than higher education to subjects with higher educational level. The differences in CFI and RMSEA for the metric and scalar models are smaller than the criteria of ΔCFI≤0.01 and ΔRMSEA≤0.015, indicating that the instrument performs similarly in different demographic groups.

**Table 3:** Analysis of invariance according to sex and educational level for the PR-COVID-19-PE.

	CFI	ΔCFI	RMSEA	ΔRMSEA
Sex				
Configural	0,974		0,039	
Metric	0,968	0,006	0,043	0,004
Scalar	0,967	0,001	0,042	0,001
Educational level				
Configural	0,981		0,034	
Metric	0,969	0,012	0,042	0,008
Scalar	0,960	0,009	0,047	0,005

CFI: Comparative Fit Index, RMSEA: Root Mean Square Error of Approximation.

### Analysis of criterion validity

Both dimensions of PR-COVID-19-PE and the total score were found to be positively, significantly and strongly related to the Dryhurst PR scale (p<0.001); but were positively, significantly and moderately related to the COVID-19 fear scale (p<0.001) (Table 4).

**Table 4:** Descriptive statistics and Pearson correlations of the scales of risk perception of COVID-19 and fear of COVID-19.

	M	SD	Pearson’s r				
			1	2	3	4	5
1. Cognitive dimension	3.76	0.51	-				
2. Emotional dimension	3.93	0.60	0.93 ^a^	-			
3. PR-COVID-19-PE	3.84	0.48	0.98 ^a^	0.99 ^a^	-		
4. Dryhurst *et al.* Perception Scale	4.40	0.83	0.68 ^a^	0.73 ^a^	0.70 ^a^	-	
5. Fear of COVID-19	2.84	0.90	0.38 ^a^	0.44 ^a^	0.41 ^a^	0.33 ^a^	-

M: mean, SD: standard deviation

a p < 0.001

## DISCUSSION

We designed and validated the PR-COVID-19-PE to measure PR before COVID-19 in a sample of Peruvian inhabitants. Our results show that this scale has adequate reliability, content, construct and criterion validity in the studied population. Likewise, it shows metric and scalar invariance according to sex and educational level.

The design of PR-COVID-19-PE was based on the multidimensional theoretical model of PR before COVID-19 proposed by Dryhurst *et al*^13^. Similarly, this model was used for the construction and validation of scales in Latin America ^15^^,^^16^. However, PR-COVID-19-PE is composed of two factors (cognitive and emotional) and therefore differs from other Latin American scales that considered items related to motivations for action ^15^ and preventive behaviors ^16^. This aspect is relevant because the PR-COVID-19-PE scale can be used to analyze its association with practices and attitudes towards preventive measures.

The instrument has content validity and is linguistically adapted because the items have a logical relationship with the dimension they are measuring (coherence, CVCic = 0.94), are relevant and important in the scale (relevance, CVCic = 0.93) and are easily understood, that is, their syntax and semantics are adequate (clarity, CVCic = 0.93). Likewise, terms that are part of the social representations of the participants, such as the variants and sequelae were incorporated in the questions thanks to the exploration made during the focus group.

The CFA showed that PR-COVID-19-PE had adequate psychometric properties in the studied sample, because all its indicators showed that the model has adequate fit in construct validity, which evidences good internal consistency. Likewise, the CFA allowed us to confirm that PR-COVID-19-PE is made up of two factors or dimensions (cognitive and emotional) that evaluate PR at a personal, family and global level; unlike other scales that only measure personal risk ^12^. The cognitive dimension comprises the subjective assessment of being infected with COVID-19 and of suffering its consequences and sequelae. This dimension ranges from the perception of the probability of infection to the self-efficacy that people have regarding the measures they adopt to prevent this viral disease. Although self-efficacy was not considered by Dryhurst *et al*. ^13^, it was nevertheless included in PR-COVID-19-PE because this construct is a mediator of preventive practices. On the other hand, the emotional dimension comprises the subjective appraisal of the severity of symptoms, of the consequences or sequelae of COVID-19 and the level of concern that these can produce.

Furthermore, the results suggest that PR-COVID-19-PE presents metric and scalar invariance by both sex and educational level. Therefore, it is appropriate to use this instrument to compare PR between men and women, as well as between individuals with different levels of education.

The criterion validity of PR-COVID-19-PE was confirmed by the finding of correlation with the two scales used for this purpose. In addition, the positive and direct correlation with the PR scale of Dryhurst *et al*. ^13^ shows that both scales measure the expected phenomenon, because they were constructed based on the theoretical framework proposed by Van der Linden ^14^. Likewise, the COVID-19 fear scale was also used to determine criterion validity during the validation of a PR scale in Italy ^11^.

Having a valid and reliable instrument that measures PR to COVID-19 from a cognitive and emotional perspective could be useful to explore how this construct varies in the complex social and psychosocial reality post-COVID-19, especially during the implementation of prevention measures such as vaccination. Likewise, it could be used to explore its association with knowledge, preventive behaviors and psychological constructs; which would lead to useful findings for the design of public policies and effective interventions in the control of new outbreaks. In addition, it will be useful to detect groups susceptible to greater COVID-19 infection compared to other possible waves, as well as to understand the determinants of risk and evaluate the most opportune moment to develop prevention behavioral change campaigns.

This psychometric study had some limitations. First, we aimed to describe the psychometric properties of the scale and used a non-probabilistic type of sampling; therefore, the results cannot be generalized to the entire Peruvian population. In addition, the information was collected with a virtual form in which participants self-reported their assessment, which may have been influenced by social desirability. This could be related to the fact that the results have a ceiling effect, so we recommend that future studies should consider broadening the range of responses.

Besides, since one of the scales used to determine criterion validity reported low reliability, it could lead to erroneous comparisons, which is why we suggest that our results regarding validity should be validated in following studies. Given the non-probabilistic sampling, the factorial model needs to be verified in future studies with different samples, even though it was based on theoretical grounds and validated by a panel of experts. The survey was conducted during the fourth wave of COVID-19 infection in Peru, so the PR of the population interviewed was probably higher than from future studies; however, PR-COVID-19-PE is an important instrument due to the cyclical evolution of epidemics and the appearance of new outbreaks. Finally, no cut-off points were evaluated to assess levels, nor their temporal stability.

In conclusion, this study designed and validated the PR-COVID-19-PE scale so that it can be used in a population with similar characteristics to the study sample. This scale, composed of two factors (cognitive and emotional), presents adequate reliability (ώ= 0.88), content and criterion validity; as well as invariance according to sex and educational level. However, additional studies in different age groups, rural populations and including other departments are recommended, as well as concurrent validity studies.
