# Supplementary material for: Development and validation of a COVID-19 risk perception scale in Peru
Source: Rev Peru Med Exp Salud Publica. 2023 Jun 30;40(2):170–8. doi: 10.17843/rpmesp.2023.402.12289 (PMC10953650; doi:10.17843/rpmesp.2023.402.12289)
Supplement: Supplementary material. — Available in the electronic version of the RPMESP. [file rpmesp-40-02-12289-s001.docx]

Material suplementario 1. Escala de percepción de riesgo ante la COVID-19

| ***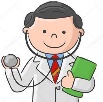INSTRUCCIÓN:***  *A continuación, se presentan una serie de enunciados sobre la percepción que tiene ante la posibilidad de contagiarse de COVID-19, cada enunciado tiene 5 opciones, marque con una (X) para representar que tan de acuerdo o en desacuerdo esta con los siguientes enunciados.* |
| --- |
|  |
| \|  \| *Totalmente en desacuerdo* \| *En desacuerdo* \| *Ni de acuerdo ni en desacuerdo* \| *De acuerdo* \| *Totalmente de acuerdo* \| \| --- \| --- \| --- \| --- \| --- \| --- \| \| 1. *Soy consciente de que aún podría contagiarme con COVID-19 y sus variantes* \|  \|  \|  \|  \|  \| \| 1. *Si completo la vacunación evitaré contagiarme con COVID-19 y sus variantes* \|  \|  \|  \|  \|  \| \| 1. *Me siento protegido de la COVID-19 y sus variantes si es que cumplo con los protocolos de prevención (uso de mascarilla, lavado de manos y distanciamiento físico).* \|  \|  \|  \|  \|  \| \|  \| ***Muy bajo riesgo*** \| ***Bajo riesgo*** \| ***Moderado riesgo*** \| ***Alto riesgo*** \| ***Muy alto riesgo*** \| \| 1. *¿Cuál considera que es el nivel de riesgo que tiene un familiar o amigo APARENTEMENTE SANO de contagiarse con COVID-19?* \|  \|  \|  \|  \|  \| \| 1. *¿Cuál considera que es el nivel de riesgo que tiene un familiar o amigo VULNERABLE de contagiarse con COVID-19? (obesidad, Hipertensión u otra enfermedad)* \|  \|  \|  \|  \|  \| \| 1. *¿Cuál considera que es el nivel de riesgo que tiene un familiar o amigo ADULTO MAYOR de contagiarse con COVID-19?* \|  \|  \|  \|  \|  \| \| 1. *¿Cuál considera que es el nivel de riesgo que tiene un niño o niña de contagiarse con COVID-19?* \|  \|  \|  \|  \|  \| \|  \| ***Totalmente en desacuerdo*** \| ***En desacuerdo*** \| ***Ni de acuerdo ni en desacuerdo*** \| ***De acuerdo*** \| ***Totalmente de acuerdo*** \| \| 1. *El riesgo de que una nueva ola de contagios por COVID-19 llegue a Perú es inminente o alta* \|  \|  \|  \|  \|  \| \| 1. *El riesgo de que aparezcan nuevas variantes de COVID-19 en el MUNDO es inminente o alta* \|  \|  \|  \|  \|  \| \| 1. *Si me contagio con COVID-19 y sus variantes podría enfermaré gravemente y requeriré hospitalización* \|  \|  \|  \|  \|  \| \| 1. *Enfermarme o quedar con secuelas a causa de la COVID-19 tendría consecuencias en mi desempeño laboral o las actividades que realizo cotidianamente* \|  \|  \|  \|  \|  \| |
|  |
| \|  \| *Totalmente en desacuerdo* \| *En desacuerdo* \| *Ni de acuerdo ni en desacuerdo* \| *De acuerdo* \| *Totalmente de acuerdo* \| \| --- \| --- \| --- \| --- \| --- \| --- \| \| 1. *Tengo miedo de contagiarme con COVID-19 y sus variantes* \|  \|  \|  \|  \|  \| \| 1. *Tengo miedo de que alguno de mis familiares se contagie con COVID-19 y sus variantes* \|  \|  \|  \|  \|  \| \| 1. *Si me contagio o alguno de mis familiares se contagia con COVID-19 tendría temor y miedo a la muerte* \|  \|  \|  \|  \|  \| \| 1. *Si me contagio con COVID-19 y sus variantes tendría el riesgo de quedar con secuelas* \|  \|  \|  \|  \|  \| \| 1. *La COVID-19 genera dolor a la familia y preocupación por los hijos y familiares.* \|  \|  \|  \|  \|  \| \| 1. *Si me contagio con COVID-19 se afectaría mi vida social.* \|  \|  \|  \|  \|  \| |
| \|  \| *Nada* \| *Un poco* \| *Bastante* \| *Mucho* \| *Muchísimo* \| \| --- \| --- \| --- \| --- \| --- \| --- \| \| 1. *¿Hasta qué punto le preocupa contagiarse o que alguno de sus familiares se contagie con COVID-19 y sus variantes?* \|  \|  \|  \|  \|  \| \| 1. *¿Hasta qué punto considera que una nueva ola de contagios por COVID-19 afectará la estabilidad económica de Perú (escases de alimentos, incrementar la pobreza y el desempleo)?* \|  \|  \|  \|  \|  \| \| 1. *¿Hasta qué punto considera que una nueva ola de contagios por COVID-19 generará crisis en el sistema de salud de Perú (colapso de hospitales o desabastecimiento de medicamentos)?* \|  \|  \|  \|  \|  \| \| 1. *¿Hasta qué punto considera que una nueva ola de contagios por COVID-19 afectará la salud mental de los peruanos (incremento de ansiedad, temor y miedo)?* \|  \|  \|  \|  \|  \| \|  \| \| \| \| \| \| \|  \| ***Totalmente en desacuerdo*** \| ***En desacuerdo*** \| ***Ni de acuerdo ni en desacuerdo*** \| ***De acuerdo*** \| ***Totalmente de acuerdo*** \| \| 1. *Una nueva ola de contagios por COVID-19 ocasionará crisis económica a nivel mundial* \|  \|  \|  \|  \|  \| |

Material suplementario 2 Checklist COSMIN (COnsensus-based Standards for the selection of health status Measurement por sus siglas en inglés)* realizado a la Escala de percepción de riesgo validada

| Nº |  | Yes | No | No aplica |
| --- | --- | --- | --- | --- |
| Internal consistency | | | | |
| 1 | Does the scale consist of effect indicatiors.i.e.is it based on a reflective model? Design requirements | X |  |  |
| 2 | Was the percentage of missing items given? | X |  |  |
| 3 | Was there a description of how missing items were handled? | X |  |  |
| 4 | Was the sample size included in the internal consistency analysis or IRT model applied? | X |  |  |
| 5 | Was the unidimensionality of the scale checked? i.e. was factor analysis or IRT model applied? |  |  | X |
| 6 | Was the sample size included in the unidimensionality analysis adequate? |  |  | X |
| 7 | Was an internal consistency statistic calculated for each(unidimensional) (sub) scale separately | X |  |  |
| 8 | Were there any important flaws in the design or methods of the study? Statistical methods | X |  |  |
| Statistical methods | | | | |
| 9 | For Classical Test Theory (CTT):Was Cronbach´s alpha calculated ? | X (coeficiente de Omega de McDonald) |  |  |
| 10 | For dichotomous scores: Was Cronbach´alpha or KR-20 calculated? |  |  | X |
| 11 | For IRT: Was a goodness of fit statistic at a global level calculated? E.g.x2. reliability coefficient of estimated lateent trait value (index of ( subject or item) separation |  |  | X |
| Content validity (including face validity)  General requirements | | | | |
| 1 | Whas there an assessment of whether all items refer to relevant aspects of the construct to be measured? | X |  |  |
| 2 | Was there an assessment of whether all items are relevant for the study population? (e.g.age,gender,disease characteristics, country,setting) | X |  |  |
| 3 | Was there an assessment of whether all items are relevant fort the purpose of the measurement instrument? (discriminative, evaluative; and/or predictive) | X |  |  |
| 4 | Was there an assessment of whether all items together comprehensively reflect the construct to be measured? | X |  |  |
| 5 | Were there any important flaws in the design or methods of the study? | X (Representatividad) |  |  |
| Hypotheses testing  Design requieremnts | | | | |
| 1 | Was the percentage of missing items given? | X |  |  |
| 2 | Was there a description of how missing items were handled? | X |  |  |
| 3 | Was the sample size included in the analysis adequate? | X |  |  |
| 4 | Were hypotheses regarding correlations or mean differences formulated a priori (i.e. before data collection)? | X |  |  |
| 5 | Was the expected direction of correlations or mean differences included in the hypotheses? | X |  |  |
| 6 | Was the expected absolute or relative magnitude of correlations or mean differences included in the hypotheses? | X |  |  |
| 7 | For convergent validity: Was an adequate description provided of the comparator instrument(s)? | X |  |  |
| 8 | For convergent validity: were the measurement properties of the comparator instrument(s) adequately described? | X |  |  |
| 9 | Were there any important flaws in the design or methods of the study?  Statistical methods |  | X |  |
| 10 | Were design and statistical methods adequate for the hypotheses to be tesded? | X |  |  |

*Fuente: Mokkink LB, Terwee CB, Knol DL, Stratford PW, Alonso J, Patrick DL, Bouter LM, de Vet HC. The COSMIN checklist for evaluating the methodological quality of studies on measurement properties: a clarification of its content. BMC Med Res Methodol. 2010;10:22. doi: 10.1186/1471-2288-10-22.

Material complementario 3. Definición de los indicadores de las dimensiones de Percepción de riesgo ante la COVID-19

| Dimensión | Indicador | Definición |
| --- | --- | --- |
| Cognitiva | Percepción de probabilidad de contagio individual | Estimación subjetiva de la posibilidad o probabilidad de que la persona se contagie de la COVID-19 en sus distintas variantes. |
|  | Percepción de autoeficacia de las medidas preventivas ante el contagio individual y familiar | Percepción de capacidad personal para el control y que al adoptar las medidas preventivas (mascarilla, lavado de manos, distanciamiento y vacunación) Y cumplir medidas evitativas logrará evitará o disminuir el riesgo de contagiarse de la COVID-19 |
|  | Percepción de probabilidad de contagio familiar o amical | Estimación subjetiva de la probabilidad de que un familiar o amigo (aparentemente sano o con factor de riesgo como ser adulto mayor o tener comorbilidad) se contagie de COVID-19 en sus distintas variantes. |
|  | Percepción de probabilidad de impacto del COVID-19 en el país | estimación subjetiva de la probabilidad de que el país se vea afectado por una ola de contagios por COVID-19 en sus distintas variantes. |
|  | Percepción de probabilidad de impacto del COVID-19 a nivel global | estimación subjetiva de la probabilidad de que el COVID-19 genere una ola de contagios a nivel mundial. |
| Emocional | Preocupación por el contagio de COVID-19 | reacción afectiva negativa ante la probabilidad de contagio de COVID-19 y posibles secuelas a nivel individual y familiar que está caracterizada por pensamientos recurrentes e invasivos, temor y miedo. |
|  | Valoración de la severidad en caso de contagio de la COVID-19 a nivel individual | valoración subjetiva del impacto en la salud personal, como padecer secuelas y repercusión en la vida frente a un contagio de COVID-19 en sus distintas variantes, así como de las posibles secuelas. Comprende también la etiqueta con la que compara la enfermedad |
|  | Valoración de la severidad en caso de contagio familiar | valoración subjetiva del impacto en la familia, salud de los familiares o amigo (aparentemente sano o con factor de riesgo como ser adulto mayor o tener comorbilidad) frente a un contagio de COVID-19 así como las posibles secuelas. |
|  | Valoración del impacto del COVID-19 en el país | valoración subjetiva del impacto económico y de salud pública ante una ola de contagios por COVID-19 en el país. |
|  | Valoración del impacto de la COVID-19 a nivel global | valoración subjetiva del impacto repercusión a nivel económico y de salud pública ante una ola de contagios por COVID-19 a nivel mundial. |
