# Supplementary material for: Development and validation of a COVID-19 risk perception scale in Peru
Source: Rev Peru Med Exp Salud Publica. 2023 Jun 30;40(2):170–8. doi: 10.17843/rpmesp.2023.402.12289 (PMC10953650; doi:10.17843/rpmesp.2023.402.12289)
Supplement: Translation

# Translation: Desarrollo y validación de una escala de percepción de riesgo de COVID-19 en Perú

**Authors:** Jhon Alex Zeladita-Huaman, Eduardo Franco-Chalco, Roberto Zegarra-Chapoñan, Ruth Iguiñiz-Romero, Isabel Amemiya-Hoshi

**Affiliations:** 1 Universidad Nacional Mayor de San Marcos, Lima, Perú.; 2 Escuela Nacional de Salud Pública - ENSAP, Lima, Perú.; 3 Universidad María Auxiliadora, Lima, Perú.; 4 Universidad Peruana Cayetano Heredia, Lima, Perú.

**Keywords:** Infecciones por Coronavirus, Percepción, Psicometría, Perú

## Abstract

**Objetivos.:**

Desarrollar y validar una escala de percepción de riesgo ante la COVID-19 (PR-COVID-19-PE) en población peruana.

**Materiales y métodos.:**

Estudio transversal psicométrico, realizado en el 2022. En la fase 1 se diseñó la escala inicial mediante revisión teórica, grupos focales, panel de expertos y revisión documental de escalas. En la fase 2 se determinó la escala mediante juicio de expertos y una prueba piloto. En la fase 3 se hizo una encuesta virtual en 678 pobladores peruanos adultos. Se efectuó un análisis factorial confirmatorio. Para determinar la validez de criterio se realizó un análisis correlacional (r de Pearson) con una escala válida de percepción de riesgo y la escala de miedo a la COVID-19.

**Resultados.:**

La PR-COVID-19-PE está compuesta por dos dimensiones (cognitivo y emocional), presentó buen ajuste en la validez de constructo (x^2^/gl=2,34, Comparative Fit Index = 0,96, Tucker-Lewis Index = 0,96, Root Mean Square Error of Approximation = 0,05 y Standarized Root Mean-Square = 0,07) y óptima consistencia interna (ώ=0,88). Asimismo, reportó correlación con otra escala de percepción de riesgo ante la COVID-19 (r=0,70, p<0,001) y miedo a la COVID-19 (r = 0,41, p < 0,001). Además, presenta invarianza métrica y escalar tanto por sexo como por nivel educativo.

**Conclusiones.:**

La escala PR-COVID-19-PE presentó adecuada confiabilidad y validez de contenido, constructo y criterio. Constituye un instrumento para medir la percepción de riesgo ante la COVID-19 en poblaciones similares. Sin embargo, se requiere estudios adicionales en diferentes grupos poblacionales.

## INTRODUCCIÓN

Al término de la pandemia por la enfermedad de coronavirus (COVID-19), se reporta más de 2,9 millones de fallecimientos a nivel mundial ^1^. En este escenario pospandémico, debido a que las vacunas fueron efectivas en disminuir la forma grave de la enfermedad y muertes ^2^, la mayoría de países han eliminado la obligatoriedad del cumplimiento de las medidas preventivas. Sin embargo, las variantes y subvariantes que aparecieron en 2021, continúan propagándose a nivel mundial, por cambios en su transmisibilidad, patogenicidad y virulencia; incluso en la manifestación de los síntomas, complicaciones y secuelas ^3^.

Por otra parte, el riesgo no es constante ni se puede reducir a una percepción de vulnerabilidad de muerte segura o de estar exento de padecer daño ^4^^,^^5^. Durante esta pandemia, la población percibió diferentes niveles de riesgo debido al incremento acelerado de fallecidos, aparición de las variantes, la incertidumbre de las secuelas y efectos a largo plazo de esta enfermedad ^6^.

Estudios realizados al inicio de la vacunación contra la COVID-19 reportaron una disminución de la percepción de riesgo (PR), sin embargo, esta percepción no se eliminará completamente ^2^. Es más, aunque estemos al término de la pandemia, no deja de ser relevante el estudio de los factores psicológicos y contextuales que dieron forma al comportamiento humano en respuesta ante un fenómeno que marcará nuestra historia, por su carácter pandémico y las consecuencias socioeconómicas y psicológicas que enfrenta la humanidad.

A pesar de que la PR también fue estudiada durante la pandemia de gripe A (H1N1) ^7^ y Ebola ^8^; se cuenta con escasa evidencia sobre su determinación, por lo que no existe consenso en cuanto a su medición. Generalmente, este constructo fue evaluado por estudios que emplean entre una a tres preguntas ^9^^-^^11^ o en breves cuestionarios de autoinforme ^11^^,^^12^. Posteriormente, un estudio transversal realizado en diez países de Europa, América y Asia validó una escala multidimensional de la PR ante la COVID-19 que comprende siete ítems que abarca las dimensiones cognitivas, afectiva y temporo-espaciales ^13^; basado en el modelo teórico de la PR ante el cambio climático propuesto por van der Linden ^14^. En el contexto latinoamericano y del Caribe se validaron dos escalas, una en Cuba ^15^ y otra en Colombia ^16^. Ambas escalas, además de considerar la dimensión cognitiva y emocional, incluyeron una dimensión que evalúa motivaciones y conductas de riesgo y protección; aspectos que podrían interferir en el análisis de asociación con otras variables como la práctica de medidas preventivas.

Sin embargo, a pesar de la existencia de escalas validadas en español ^15^^,^^16^ para medir la PR ante la COVID-19, estudios en Perú emplearon escalas traducidas de otros idiomas ^17^, adaptadas de cuestionarios empleados en otras enfermedades como el cáncer ^18^ o mediante una sola pregunta ^9^. En otro estudio, realizado en personal de salud, se empleó una escala de percepción de seguridad acerca de las medidas de protección ^19^ Por estas razones, es necesario contar con una escala que realmente mida lo que se desea medir en la población peruana; más aún si se tiene en cuenta que esta se caracteriza por presentar una amplia diversidad cultural ^20^.

Así mismo, si bien el inicio de la vacunación contra la COVID-19 conllevó a que la población perciba menor riesgo sobre la severidad de la enfermedad. No obstante, el poder usar una escala para identificar como diferentes factores contextuales y psicológicos hacen que dicha percepción cambie de un momento al siguiente, es precisamente el aspecto que justifica el desarrollo de una escala para medir este constructo. Aunado a ello, la PR varía según diferencias culturales, factores sociales, psicológicos y políticos ^13^; así como, por la intensidad de los efectos o consecuencias del SARS-CoV-2 en una determinada población ^21^. Por estos motivos se realizó el presente estudio que tuvo como objetivo desarrollar y validar una escala de PR de COVID-19 en población peruana.

**MENSAJES CLAVE**

Motivación para realizar el estudio. La percepción de riesgo ante la COVID-19 es un constructo que varía según las características de la población de cada área geográfica; sin embargo, no existe una escala validada para medir este constructo en población peruana.

Principales hallazgos. Se diseñó y validó una escala de percepción de riesgo ante la COVID-19 compuesta por dos dimensiones (cognitiva y emocional) mediante técnicas cualitativas y cuantitativas.

Implicancias. Contar con un instrumento válido y confiable permitirá identificar la variación de la percepción de riesgo ante la COVID-19 según factores contextuales y psicológicos en la población peruana.

## MATERIALES Y MÉTODOS

### Diseño del estudio

Se desarrolló un estudio no experimental, transversal y de tipo instrumental ^22^ que consistió en la elaboración, validación y determinación de las propiedades psicométricas de una escala de PR ante la COVID-19 en población peruana. Cabe señalar que, en el periodo de realización del estudio, Perú se encontraba iniciando la cuarta ola de contagios por la COVID-19 ^23^.

### Instrumento

Escala de PR ante la COVID-19 (PR-COVID-19-PE) desarrollado en el presente estudio (Material suplementario 1).

### Procedimientos

Para verificar la adecuación de los procedimientos de análisis de validez del instrumento, se utilizó el COSMIN Checklist (COnsensus-based Standards for the selection of health status Measurement, por sus siglas en inglés) (https://www.cosmin.nl/) (Material suplementario 2).

El presente estudio tuvo tres etapas organizadas con base en los diez pasos propuestos por Muñiz y Fonseca ^24^. La primera etapa fue la orientada al diseño de la escala, para lo cual se realizó la revisión teórica sobre PR en general y PR de la COVID-19 para delimitar el marco teórico y definir la variable, dimensiones e indicadores. Luego, un panel de nueve expertos (autores de artículos científicos sobre validación de escalas de PR ante la COVID-19 en países latinoamericanos o que hicieron estudios sobre prevención de COVID-19 en Perú) validaron estas definiciones y brindaron sugerencias que fueron incorporadas en las siguientes etapas del estudio.

Posteriormente, para la construcción de los ítems, se conformaron seis grupos focales que exploraron las representaciones sociales sobre la PR de la COVID-19 que tienen los diferentes segmentos de la población peruana. Los grupos focales estuvieron conformados por docentes de educación básica regular (nivel primaria y secundaria), profesores de educación superior, estudiantes de educación superior, padres de familia y comerciantes; quienes brindaron su consentimiento informado previo a la sesión. Mediante el programa Atlas.Ti v.8 se transcribieron y codificaron las declaraciones de los participantes de los grupos focales.

Luego, se realizó una revisión documental de las escalas de PR ante la COVID-19 validadas a la fecha ^13^^,^^15^^,^^16^^,^^25^. Se realizó la triangulación de datos considerando las declaraciones de los participantes en los grupos focales, los indicadores elaborados en la revisión teórica y la información de la revisión documental. Como producto se construyó la propuesta de escala inicial compuesta por 26 ítems, sus dimensiones y sus alternativas de respuesta.

En la segunda etapa del estudio, para estimar la validez de contenido, se realizó un juicio de expertos (dos psicólogos psicométricos, un psicólogo neurocientífico y dos enfermeras investigadoras en salud pública) a quienes se les envió los documentos conteniendo las definiciones teóricas y la escala de PR propuesta junto con su escala de calificación. La calificación de los expertos fue consolidada y mediante el índice de concordancia del Coeficiente de Validez de Contenido (CVCic) de Hernández Nieto ^26^, se evaluaron tres criterios: coherencia, relevancia y claridad.

Simultáneamente, para evaluar la presentación de la escala y detectar los ítems que podrían presentar dificultades en su comprensión y errores en su formulación, se efectuó una prueba piloto en 41 pobladores de trece ciudades de Perú, a quienes vía telefónica se les invitó a completar un formulario virtual diseñado en Google Forms que fue difundido por WhatsApp. Posteriormente, se les solicitó completar un formulario adicional para reportar alguna dificultad en la compresión de los ítems y las alternativas de respuesta.

Por último, la tercera etapa de este estudio se orientó a determinar las propiedades psicométricas del instrumento diseñado. Inicialmente, se calculó un tamaño de muestra mínimo de 260, asumiendo el criterio clásico de 10 sujetos por cada variable del modelo ^27^; sin embargo, para tener una mayor potencia estadística se incluyeron a los 678 participantes que respondieron la encuesta. La muestra se seleccionó mediante muestreo no aleatorio por bola de nieve.

Entre el 5 al 21 de julio del 2021, se invitó a participar a los peruanos mayores de 18 años. La recopilación de datos se realizó mediante un formulario virtual diseñado en la plataforma ALLCOUNTED (https://www.allcounted.com), que fue difundido por redes sociales y en la página web de la Escuela Nacional de Salud Pública del Ministerio de Salud.

### Variable

La PR ante la COVID-19 fue definida para esta investigación como la valoración subjetiva de la probabilidad de contagio y severidad de las posibles consecuencias de la COVID-19 a nivel individual, familiar y global. Este constructo está conformado por dos dimensiones, una cognitiva y otra emocional. Las definiciones de los indicadores se presentan en el Material suplementario 3.

### Análisis estadístico

En el procesamiento estadístico para corroborar la estructura factorial hipotetizada de la escala se realizó un análisis factorial confirmatorio (AFC) que permitió evaluar la estructura factorial de dos dimensiones. Este estudio no realizó el análisis factorial exploratorio debido a que se tenía definida una estructura teórica planteada desde el marco conceptual y el análisis cualitativo, siendo el AFC una estrategia adecuada en estas situaciones ^28^^,^^29^. Debido a la naturaleza ordinal del instrumento, se utilizó un estimador de mínimos cuadrados de medias y varianzas ponderadas (Weigted Least Squares Means and Variances). Para identificar el ajuste absoluto del modelo se procedió a revisar si la razón de chi cuadrado entre los grados de libertad (X_2_/ gl) era menor a 3. Del mismo modo para evaluar el ajuste relativo se verificó si los indicadores de Comparative Fit Index (CFI, por sus siglas en inglés) y Tucker-Lewis Index (TLI, por sus siglas en inglés) eran mayores a 0,95, al igual que si el indicador Root Mean Square Error of Approximation (RMSEA, por sus siglas en inglés) era igual o menor a 0,05 y el indicador Standarized Root Mean-Square (SRMR, por sus siglas en inglés) igual o menor a 0,08 ^30^^-^^32^. En este paso se consideró la opción de eliminar ítems que no tuvieran una carga factorial significativa o con niveles muy bajos. Del mismo modo, se realizó un análisis de invarianza según sexo y el nivel educativo para comparar la invarianza configural, métrica y escalar, para este criterio se consideraron cambios menores o iguales en 0,01 en CFI ^33^, y cambios menores o iguales en 0,015 en RMSEA ^34^ para considerar alcanzado el nivel de invarianza.

La consistencia interna se determinó mediante el coeficiente de omega de McDonald (ώ). Para verificar la validez de criterio de la PR-COVID-19-PE se utilizaron como medida de contraste a dos cuestionarios. Primero se contrastó con un cuestionario validado que mide PR ante la COVID-19 ^13^, que fue seleccionado debido a que emplea el modelo teórico sobre el que se construyó la escala ^14^, sin embargo, debido a la baja confiabilidad (ώ=0,53) se eliminó el ítem 6 que mostraba no estar correlacionado con los otros ítems del instrumento; logrando así incrementar el valor del índice de confiabilidad (ώ=0,68 de la encuesta). El segundo contraste con el cuestionario de miedo al COVID-19 ^35^ presentó una alta confiabilidad (ώ=0,93). Ambos instrumentos fueron correlacionados con PR-COVID-19-PE, con el coeficiente de correlación r de Pearson. En cuanto a los datos perdidos, se identificó un 7,4% de información perdida, se realizó la prueba de Little, prueba que contrasta la hipótesis nula de valores perdidos completamente aleatorios, donde no se halló suficiente evidencia para rechazar que la pérdida de datos era *missing completely at random* (MCR, por sus siglas en inglés), por lo que se procedió a realizar imputación múltiple con 20 bases de datos y el algoritmo Predictive Mean Matching (PMM, por sus siglas en inglés). Se realizaron revisiones de gráficos de cuantil-cuantil y pruebas de Shapiro-Wilk para determinar el nivel de normalidad de las variables, donde se pudo observar que la distribución de los datos cumple un criterio suficiente para considerar que provienen de una distribución normal. Por lo que se usaron pruebas paramétricas para identificar las asociaciones de la validez concurrente. Todos los análisis estadísticos del presente estudio se efectuaron en el software R v 4.2.1 y su interfaz R Studio v 2021.0. 

### Aspectos éticos

El artículo deriva del estudio titulado: «Percepción de riesgo y prácticas de medidas preventivas ante la COVID-19 en docentes de Educación Básica Regular, Perú, 2022: Un estudio mixto» que fue aprobado por el Comité de Ética e Investigación de la Universidad María Auxiliadora (Acta n.° 004-2022) y registrado (código EI00000002729) en la Plataforma de Proyectos de Investigación en Salud (PRISA) del Instituto Nacional de Salud (INS). Los participantes dieron su consentimiento informado previo a su participación en el estudio y no se recolectaron datos que permitan identificarlos. 

## RESULTADOS

En los grupos focales participaron 29 pobladores de 11 ciudades de Perú, de las cuales 17 fueron mujeres (58,6%) y la edad promedio fue de 41,07 años (DE=14,19). El 58,6% de participantes reportaron tener estudios superiores completos, el 13,7% estudios superiores incompletos, mientras que el 27,6% solo cursaron secundaria completa. Asimismo, en la prueba piloto participaron 41 personas, de los cuales el 70% fueron de sexo femenino y el 36,6% radican en Lima.

### Validez de la escala

El CVCic obtenido para cada uno de los ítems evaluados en el juicio de expertos se reporta en la Tabla 1, donde se pueden observar que para la mayoría fue mayor de 0,90. Asimismo, el CVCic obtenido para los criterios de coherencia, relevancia y claridad fue mayor de 0,90.

**Tabla 1:** Coeficientes de validez de contenido para los criterios de coherencia, relevancia y claridad por ítem de la escala inicial de percepción de riesgo ante la COVID-19.

Ítem	Coherencia	Relevancia	Claridad
1	0,99	0,99	0,96
2	0,96	0,99	0,96
3	0,99	0,99	0,96
4	0,83	0,75	0,96
5	0,88	0,83	0,99
6	0,99	0,92	0,83
7	0,99	0,96	0,99
8	0,96	0,92	0,96
9	0,96	0,92	0,99
10	0,92	0,88	0,99
11	0,99	0,96	0,99
12	0,96	0,92	0,99
13	0,99	0,99	0,99
14	0,99	0,99	0,99
15	0,96	0,92	0,99
16	0,75	0,71	0,92
17	0,83	0,92	0,99
18	0,88	0,92	0,99
19	0,83	0,96	0,99
20	0,92	0,92	0,99
21	0,99	0,99	0,99
22	0,99	0,99	0,99
23	0,96	0,96	0,96
24	0,96	0,96	0,96
25	0,99	0,99	0,99
26	0,92	0,88	0,99
CVCic por criterio	0,94	0,93	0,98

CVCic = índice de concordancia del coeficiente de validez de contenido.

En la prueba piloto se determinó que el tiempo promedio de aplicación del cuestionario fue 12 min. De los 41 participantes de la prueba piloto, 34 aceptaron completar el segundo formulario mediante una entrevista. En esta etapa, solo 6 de los 34 reportaron haber tenido alguna dificultad en responder los ítems.

Finalmente, se evaluaron todos los ítems que reportaban CVCic menor de 0,90 en el juicio de expertos y los que reportaron dificultades en la prueba piloto. En esta evaluación se reformularon 9 ítems (2, 3, 7, 8, 9,10,16,17 y 20), se cambiaron 3 ítems a otra dimensión (17, 18 y 19) y se eliminaron 3 ítems (4, 5 y 6); por lo que la escala quedó conformada por 23 ítems.

### Análisis factorial confirmatorio

La encuesta virtual para realizar el AFC la respondieron 780 participantes, de los cuales se excluyeron cinco por control de calidad y 97 porque contestaron solo la primera parte del cuestionario. Del total de 678 participantes, la mayoría fueron mujeres (67,0%), la edad promedio de 34,5 años (DE= 12,41). El 50,8% de la muestra reportó tener al menos estudios superiores, un 24,3% reportó tener estudios superiores incompletos, mientras que el resto de los participantes reportaron niveles inferiores de instrucción. Con respecto a la experiencia previa con la COVID-19, el 59,2% de los participantes reportaron haberse infectado previamente y un 2,5% reportó tener el diagnóstico de COVID-19 al momento de responder la encuesta. Por otro lado, el 34,3% de los participantes reportaron haber perdido un familiar o persona cercana a causa de la COVID-19.

Inicialmente, al considerar todos los ítems se obtuvo como indicadores de ajuste: X^2^/gl=3,61, CFI=0,92, TLI= 0,91, RMSEA=0,07 y SRMR=0,09. En este punto, se detectó que el ítem de codificación inversa «Si me contagio con COVID-19 será como una simple gripe o algo pasajero, luego podre continuar con mi vida» no tenía una carga factorial significativa que permitiera un ajuste adecuado del modelo hipotético utilizado, se procedió a eliminar este ítem del modelo. Del mismo modo, se dejaron libres las correlaciones residuales de los ítems con una redacción similar (pe. Ítems 4, 5, 6 y 7; y ítems 19, 20, 21, 22). Finalmente, los indicadores de ajuste sin considerar el ítem de codificación inversa fueron: X^2^/gl=2,34, CFI=0,96, TLI=0,96, RMSEA=0,05 y SRMR = 0,07. El modelo factorial final probado es el que se observa en la figura 1, el cual quedó finalmente conformado por 22 ítems.

**Figura 1:** Modelo factorial para la escala de percepción de riesgo de la COVID-19.

En la Tabla 2, se pueden observar los promedios y desviaciones estándar para cada uno de los ítems de PR-COVID-19-PE. En general, el ítem 17 obtuvo el mayor puntaje promedio (M=4,21, DE=0,69), mientras que el ítem 4 obtuvo el menor puntaje promedio (M=3,15, DE=0,90). Dado que todos los ítems fueron medidos en una escala de 1 a 5, se puede observar que los promedios de todos los ítems están por encima de 3, esto indicaría que en general las personas que participaron en el estudio tienen una percepción de riesgo superior al valor promedio de tres.

**Tabla 2:** Promedio y desviación estándar por ítems del Cuestionario de Percepción de Riesgo de la COVID-19.

Ítem	M	DE
1. Soy consciente de que aún podría contagiarme con COVID-19 y sus variantes	4,20	0,99
2. Si completo la vacunación evitaré contagiarme con COVID-19 y sus variantes	3,18	1,25
3. Me siento protegido de la COVID-19 y sus variantes si es que cumplo con los protocolos de prevención	3,96	1,00
4. ¿Cuál considera que es el nivel de riesgo que tiene un familiar o amigo aparentemente sano de contagiarse con COVID-19?	3,15	0,96
5. ¿Cuál considera que es el nivel de riesgo que tiene un familiar o amigo vulnerable de contagiarse con COVID-19?	4,13	0,90
6. ¿Cuál considera que es el nivel de riesgo que tiene un familiar o amigo adulto mayor de contagiarse con COVID-19?	4,19	0,83
7. ¿Cuál considera que es el nivel de riesgo que tiene un niño o niña de contagiarse con COVID-19?	3,36	1,14
8. El riesgo de que una nueva ola de contagios por COVID-19 llegue a Perú es inminente o alta	4,05	0,83
9. El riesgo de que aparezcan nuevas variantes de COVID-19 en el MUNDO es inminente o alta	4,07	0,83
10. Tengo miedo de contagiarme con COVID-19 y sus variantes.	3,66	1,05
11. Tengo miedo de que alguno de mis familiares se contagie con COVID-19 y sus variantes	4,02	0,89
12. Si me contagio o alguno de mis familiares se contagia con COVID-19 tendría temor y miedo a la muerte	3,71	1,01
13. Si me contagio con COVID-19 y sus variantes tendría el riesgo de quedar con secuelas	3,84	0,86
14. Si me contagio con COVID-19 y sus variantes podría enfermarme gravemente y requeriré hospitalización	3,36	1,02
15. Enfermarme o quedar con secuelas a causa de la COVID-19 tendría consecuencias en mi desempeño laboral o las actividades que realizo cotidianamente	3,67	0,93
16. La COVID-19 genera dolor a la familia y preocupación por los hijos y familiares.	4,21	0,69
17. Si me contagio con COVID-19 se afectaría mi vida social	3,57	1,02
18. ¿Hasta qué punto le preocupa contagiarse o que alguno de sus familiares se contagie con COVID-19 y sus variantes?	3,83	1,07
19. ¿Hasta qué punto considera que una nueva ola de contagios por COVID-19 afectará la estabilidad económica de Perú (escases de alimentos, incrementar la pobreza y el desempleo)?	4,08	0,90
20. ¿Hasta qué punto considera que una nueva ola de contagios por COVID-19 generará crisis en el sistema de salud de Perú (colapso de hospitales o desabastecimiento de medicamentos)?	4,08	0,94
21. ¿Hasta qué punto considera que una nueva ola de contagios por COVID-19 afectará la salud mental de los peruanos (incremento de ansiedad, temor y miedo)?	4,02	0,90
22. Una nueva ola de contagios por COVID-19 ocasionará crisis económica a nivel mundial	4,14	0,75

M: media, DE: desviación estándar.

La estructura factorial resultante del modelo confirmatorio, las cargas factoriales y sus respectivas significancias se presentan en la Figura 1. Todos los ítems de la dimensión cognitiva de PR-COVID-19-PE tienen cargas factoriales estadísticamente significativas (p<0,001), excepto en el ítem 2 (p=0,001) e ítem 3 (p=0,003). Sin embargo, es importante resaltar que los ítems 1, 2 y 3 tienen cargas factoriales con tamaños de efecto bajo (0,28, 0,16 y 0,15, respectivamente). Esto señala que los tres ítems tienen una carga significativa, pero no se encuentran tan fuertemente correlacionados con la dimensión cognitiva; sin embargo, se conservaron debido a que se considera que permiten explicar algunas características de la dimensión cognitiva que otros ítems no serían capaces de realizar. Todos los otros ítems de dicha dimensión tienen cargas factoriales superiores a 0,30 lo cual indica que tienen una buena correlación con el factor de la dimensión cognitiva. Todos los ítems de la dimensión emocional tienen cargas factoriales mayores a 0,30 y son estadísticamente significativas (p<0,001). Además, se puede observar una correlación muy fuerte entre ambas dimensiones de la escala (r=0,87, p<0,001). Finalmente, se corrobora que PR-COVID-19-PE presenta un excelente coeficiente de consistencia interna (ώ=0,88).

En la Tabla 3 se presentan los resultados de los análisis de invarianza según sexo y nivel educativo. Para el nivel educativo se compararon los sujetos con nivel educativo por debajo del superior en comparación con los sujetos con un nivel educativo superior. Las diferencias en CFI y RMSEA para los modelos métricos y escalares son menores que los criterios de ΔCFI≤0,01 y ΔRMSEA≤0,015, lo que indica que el instrumento funciona de manera similar en diferentes grupos demográficos.

**Tabla 3:** Análisis de invarianza según sexo y nivel educativo para el PR-COVID-19-PE.

	CFI	ΔCFI	RMSEA	ΔRMSEA
Sexo				
Configural	0,974		0,039	
Métrica	0,968	0,006	0,043	0,004
Escalar	0,967	0,001	0,042	0,001
Nivel educativo				
Configural	0,981		0,034	
Métrica	0,969	0,012	0,042	0,008
Escalar	0,960	0,009	0,047	0,005

CFI: Comparative Fit Index, RMSEA: Root Mean Square Error of Approximation.

### Análisis de validez de criterio

Ambas dimensiones de PR-COVID-19-PE y el puntaje total se encuentran positiva, significativa y fuertemente relacionadas a la escala de PR de Dryhurst (p<0,001); pero se encuentran positiva, significativa y moderadamente relacionada con la escala de miedo al COVID-19 (p<0,001) (Tabla 4).

**Tabla 4:** Estadísticos descriptivos y correlaciones de Pearson de las escalas de percepción de riesgo al COVID-19 y miedo al COVID-19.

	M	DE	r de Pearson				
			1	2	3	4	5
1. Dimensión cognitiva	3,76	0,51	-				
2. Dimensión emocional	3,93	0,60	0,93 ^a^	-			
3. PR-COVID-19-PE	3,84	0,48	0,98 ^a^	0,99 ^a^	-		
4. Escala de Percepción de Dryhurst *et al.*	4,40	0,83	0,68 ^a^	0,73 ^a^	0,70 ^a^	-	
5. Miedo a la COVID-19	2,84	0,90	0,38 ^a^	0,44 ^a^	0,41 ^a^	0,33 ^a^	-

M: media, DE: desviación estándar

a p < 0,001

## DISCUSIÓN

Esta investigación instrumental en la cual se diseñó y validó PR-COVID-19-PE para medir la PR ante la COVID-19 en una muestra de pobladores peruanos, reporta como resultados que esta escala presenta adecuada confiabilidad y validez de contenido, constructo y criterio en la población estudiada. Asimismo, presenta invarianza métrica y escalar según sexo y nivel educativo.

El diseño de PR-COVID-19-PE estuvo basado en el modelo teórico multidimensional de PR ante la COVID-19 propuesta por Dryhurst *et al.*^13^. De manera similar, este modelo fue empleado en la construcción y validación de escalas a nivel latinoamericano ^15^^,^^16^. Sin embargo, PR-COVID-19-PE está conformado por dos factores (cognitivo y emocional) por lo que difiere de otras escalas latinoamericanas que consideraron ítems relacionados con motivaciones para acción ^15^ y conductas preventivas ^(^^16^. Este aspecto cobra relevancia debido a que la escala PR-COVID-19-PE puede ser empleada para analizar su asociación con prácticas y actitudes hacia las medidas preventivas.

El instrumento presenta validez de contenido y se encuentra adaptado lingüísticamente debido a que los ítems presentan una relación lógica con la dimensión que están midiendo (coherencia, CVCic = 0,94), son relevantes e importantes en la escala (relevancia, CVCic = 0,93) y se comprenden fácilmente, es decir su sintáctica y semántica son adecuados (claridad, CVCic = 0,93). Asimismo, la exploración mediante el grupo focal permitió incorporar en la formulación de las preguntas términos que forman parte de las representaciones sociales de los participantes, como: variantes y secuelas.

Los resultados del AFC evidencian que PR-COVID-19-PE tiene adecuadas propiedades psicométricas en la muestra estudiada debido a que todos sus indicadores señalan que el modelo tiene adecuado ajuste en la validez de constructo que evidencia una buena consistencia interna. Asimismo, el AFC permitió sostener que PR-COVID-19-PE está conformada por dos factores o dimensiones (cognitiva y emocional) que evalúan la PR a nivel personal, familiar y global; a diferencia de otra escala que solo miden el riesgo personal ^12^. La dimensión cognitiva comprende la valoración subjetiva de contagiarse de COVID-19 y de sufrir sus consecuencias y secuelas. Esta dimensión abarca desde la percepción de la probabilidad de contagio hasta la autoeficacia que tienen las personas respecto de las medidas que adoptan para prevenir esta enfermedad viral. Aunque, la autoeficacia no fue considerada por Dryhurst *et al*.; ^13^ sin embargo, fue incluida en PR-COVID-19-PE porque este constructo es un mediador con las prácticas preventivas. Por otra parte, la dimensión emocional comprende la valoración subjetiva de la severidad de los síntomas, de las consecuencias o secuelas de la COVID-19 y el nivel de preocupación que estas generan.

Aunado a ello, los resultados sugieren que PR-COVID-19-PE presenta invarianza métrica y escalar tanto por sexo como por nivel educativo. Por lo tanto, es apropiado utilizar este instrumento para comparar la PR entre hombres y mujeres, así como entre individuos con diferentes niveles de instrucción.

La validez de criterio de PR-COVID-19-PE se corroboró con el hallazgo de la correlación con las dos escalas empleadas para este fin. Además, la correlación positiva y directa con la escala de PR de Dryhurst *et al.*, ^13^ evidencia que ambas escalas miden el fenómeno esperado debido a que fueron construidas con base en el marco teórico propuesto por Van der Linden ^14^. Asimismo, la escala de miedo a la COVID-19 también fue empleada para determinar la validez de criterio durante la validación de una escala de PR en Italia ^11^.

Contar con un instrumento válido y confiable que mide la PR ante la COVID-19 desde una perspectiva cognitiva y emocional puede ser de utilidad para explorar como varía este constructo en la compleja realidad social y psicosocial en tiempos de pos-COVID-19, en especial durante la implementación de medidas de prevención como es la vacunación. Asimismo, podría ser utilizado para explorar su asociación con conocimientos, comportamientos preventivos y constructos psicológicos; que conllevaría a obtener hallazgos útiles para el diseño de políticas públicas e intervenciones eficaces en el control de nuevos brotes. Además, servirá para detectar grupos susceptibles de mayor contagio por COVID-19 frente a otras olas posibles, así como para la comprensión de los determinantes de los riesgos y evaluar el momento más oportuno para desarrollar campañas de cambios conductuales de prevención.

Este estudio psicométrico presentó algunas limitaciones. Primero, debido a que el interés del estudio fue describir las propiedades psicométricas de la escala, se empleó un tipo de muestreo no probabilístico, por lo que los resultados no se pueden generalizar a toda la población peruana. Segundo, la información fue recopilada mediante un formulario virtual en el cual los participantes autorreportaron su valoración, el cual puede haber estado influenciado por la deseabilidad social. Esto se relacionaría al hecho de que se tienen resultados de efecto techo, por lo que se recomienda que futuros estudios consideren ampliar el rango de respuestas del instrumento.

Tercero, debido a que una de las escalas empleadas para determinar la validez de criterio reportó baja confiabilidad podría generar comparaciones erróneas, motivo por el cual se sugiere en estudios posteriores corroborar la validez hallada en este estudio. Cuarto, dado el muestreo no probabilístico, el modelo factorial, por más que fuera sustentado en bases teóricas y validado por un panel de expertos, es necesario que se confirme en futuros estudios con distintas muestras. La encuesta fue realizada durante la cuarta ola de contagios por COVID-19 en Perú, por lo que la PR de la población entrevistada probablemente ha sido mayor que la que se obtendrá en estudios posteriores, sin embargo, PR-COVID-19-PE es un instrumento importante de medida debido a la evolución cíclica de las epidemias y la aparición de nuevos brotes. Finalmente, no se evaluaron puntos de corte para evaluar niveles, ni su estabilidad temporal.

En conclusión, este estudió diseñó y validó la escala PR-COVID-19-PE para que pueda ser empleada en población con similares características a la muestra del estudio. Esta escala compuesta por dos factores (cognitivo y emocional) presenta adecuada confiabilidad (ώ= 0,88), validez de contenido y criterio; así como invarianza según sexo y nivel de instrucción. Sin embargo, se recomienda estudios adicionales en diferentes grupos etarios, poblaciones rurales y que incluyan otros departamentos; así como, estudios de validez concurrente.
